# Platelet rich plasma injections for knee osteoarthritis: an overview of systematic reviews

**DOI:** 10.3389/fphys.2025.1598514

**Published:** 2025-07-02

**Authors:** Lin Yi, Fei Qiu, Haiyuan Song, Huiyuan Huang, Guanghui Zhang

**Affiliations:** ^1^ Department of Engineering Research Center, The First Affiliated Hospital of Hunan University of Medicine, Huaihua, Hunan, China; ^2^ Department of Medical Technology, Hunan Primary Digital Engineering Technology Research Center for Medical Prevention and Treatment, Huaihua, Hunan, China; ^3^ Department of Traditional Chinese Medicine, Hunan University of Chinese Medicine, Changsha, Hunan, China; ^4^ Department of Medical Technology, Hunan Engineering Research Center of TCM Real-World Clinical Practice, Huaihua, Hunan, China

**Keywords:** platelet-rich plasma, knee osteoarthritis, AMSTAR2, groove, ROB

## Abstract

**Introduction:**

Knee osteoarthritis (KOA) is a prevalent degenerative joint disease, particularly affecting the aging population. While numerous systematic reviews (SRs) and meta-analyses (MAs) have evaluated the efficacy and safety of platelet-rich plasma (PRP) for KOA, the methodological quality and potential biases of these syntheses require critical assessment.

**Methods:**

We conducted an overview of SRs/MAs on PRP for KOA. Comprehensive searches were performed in PubMed, Embase, The Cochrane Library, and Web of Science from inception to December 1, 2024. Two reviewers independently screened literature and extracted data. The methodological quality of included SRs/MAs was evaluated using AMSTAR-2, the degree of primary study overlap was assessed using the GROOVE tool, and the risk of bias in the primary randomized trials was evaluated using the ROB 2.0 tool.

**Result:**

A total of 29 SRs/MAs met the inclusion criteria. GROOVE analysis revealed a very high degree of overlap among the primary studies included across the reviews. AMSTAR-2 assessment demonstrated critically low methodological quality for 26 reviews and low quality for the remaining 3 reviews.

**Discussion:**

The current quality of SRs/MAs on PRP for KOA remains suboptimal. Future studies should adhere closely to established evaluation frameworks including AMSTAR2 to enhance research reliability and clinical applicability.

**Systematic Review Registration:**

https://www.crd.york.ac.uk/PROSPERO/, identifier CRD42024619416.

## 1 Introduction

Knee osteoarthritis (KOA) represents the most common subtype of osteoarthritis, primarily marked by the gradual deterioration and breakdown of articular cartilage (Katz et al., 2021). Its pathological characteristics encompass cartilage degradation, alterations in subchondral bone architecture, development of osteophytes, and synovial membrane inflammation, among other changes (Weber et al., 2021). With the progressive aging of the global population, the annual incidence of KOA continues to rise. According to epidemiological data, the global incidence of KOA in patients over 40 years old is approximately 22.9%. Projections indicate that by 2050, the number of cases is expected to increase by 74.9% compared to 2020, which will impose a substantial economic burden on society and families (Cui et al., 2020; GBD, 2021 Osteoarthritis Collaborators, 2023). The knee’s anatomical characteristics, including its avascular and innervated nature, contribute to its limited capacity for self-regeneration following injury, thereby rendering the treatment of KOA a challenging endeavor (Zhang et al., 2024). In the early and intermediate phases of clinical intervention, conservative management remains the mainstay, including the application of non-steroidal anti-inflammatory drugs (NSAIDs), hyaluronic acid (HA) injections, and therapeutic exercises. However, NSAIDs are linked to gastrointestinal side effects, while joint replacement procedures are associated with various complications. Intra-articular drug injection therapy offers certain advantages in relieving patient symptoms (Bannuru et al., 2019; Kolasinski et al., 2020).

Platelet-rich plasma (PRP) is known to be enriched with bioactive molecules and proteins, such as platelet-derived growth factors, transforming growth factor beta (TGF-β), and vascular endothelial growth factor. These constituents can facilitate the migration, proliferation, and differentiation of autologous cells, accelerate the healing of tissues with limited regenerative capacity, and support the restoration of damage associated with aging (Dhillon et al., 2019; Dóri et al., 2021). The safety of PRP is attributable to its natural origin, which also minimizes the potential for adverse effects. Consequently, PRP has gained widespread application in diverse medical disciplines, including articular cartilage repair, tendon and ligament repair, and wound repair (Lai et al., 2023). Currently, a growing number of systematic reviews (SRs) and meta-analyses (MAs) are being conducted to evaluate and compare the therapeutic effectiveness and safety profiles of PRP and other agents injection in the treatment of KOA. The credibility of these studies in guiding clinical decision-making largely depends on the methodological rigor of the SRs/MAs. However, A comprehensive collection of evidence is necessary to re-evaluate the systematic review. This study undertakes a comprehensive evaluation of the SRs/MAs concerning PRP in the context of treating KOA. To evaluate the methodological soundness, study overlap and potential bias of the included SRs/MAs, this study employs the AMSTAR2 checklist, GROOVE tool and ROB2.0 tools, thus offering a reference framework for clinical application.

## 2 Materials and methods

### 2.1 Protocol registration

The study protocol was prospectively deposited in PROSPERO (registration ID: CRD42024619416) prior to commencement. Public access to the registered protocol is available at: https://www.crd.york.ac.uk/PROSPERO/.

### 2.2 Database and search strategy

A systematic search of publications was performed across PubMed, Web of Science, Embase and Cochrane Library databases through 1 December 2024. In addition, the references retrieved from the SRs/MAs should be manually supplemented and, where possible, gray literature searched to increase the completeness of the search. See Supplementary Appendix 1 in Supplement for the search strategy.

### 2.3 Inclusion and exclusion criteria

SRs/MAs of randomized controlled trials (RCTs) were selected using PICOS criteria. Population (P): Clinically diagnosed KOA patients, with no restrictions on demographics, comorbidities, or Kellgren-Lawrence grading. Intervention/Comparison (I/C): the included SRs/MAs had compared all types of PRP with the following agents injection: HA, BMAC, and other pharmacological agents. Outcomes (O): Required ≥2 validated metrics from: WOMAC, VAS, IKDC, KOOS, EQ-VAS, Lequesne index, or adverse event reporting. Study design (S): The SRs/MAs of randomized controlled trials. Dosage, preparation protocols, injection frequency were unrestricted.

Exclusion Criteria: PRP used in hand, hip, ankle and other joints with OA; duplicate publications; incomplete data; literature for which the full text could not be obtained were excluded; the design schemes of SRs/MAs were also excluded.

### 2.4 Literature review and data extraction

Two researchers (L.Y., H-Y.S.) independently conducted the literature search and used Endnote to remove duplicates. Obviously unqualified records were first filtered through titles and abstracts evaluation, followed by full-text retrieval and appraisal of retained publications. A predefined extraction template was applied to systematically collect data. The following parameters were collected: information such as author, year and place of publication, number of studies included in the literature, sample size, study type, intervention, methodological quality assessment tool, and outcome indicators. Two researchers (L.Y., H-Y.S.) cross-checked the results of the extraction and, in case of disagreement, discussed them with the arbitrator (F.Q.) to reach a decision.

### 2.5 Methodological quality assessment

The methodological quality of the included SRs/MAs was evaluated utilizing the AMSTAR-2 tool. AMSTAR-2 contains 16 items, of which items 2, 4, 7, 9, 11, 13, and 15 are key items. Each items was evaluated as yes, no or partly. The methodological quality is rated as high, moderate, low, or Critically low according to the results of the key items and non-key items (Shea et al., 2017). The evaluation was conducted independently by two researchers (L.Y., H-Y.S.). In case of disagreement, the decision shall be made after discussion with the arbitrator (F.Q.).

### 2.6 Study overlap assessment

Study overlap was measured using the GROOVE tool (Pérez-Bracchiglione et al., 2022), calculating corrected coverage area (CCA) and categorizing overlap as slight (CCA <5%), moderate (5%–10%), high (10%–15%), or very high (≥15%). When original studies were highly overlapping, we excluded duplicate studies from the SRs/MAs and assessed bias using ROB 2.0 (Sterne et al., 2019).

### 2.7 Statistical analysis

We analyzed several different knee function scales, including WOMAC, VAS and IKCD, and others. However, given the similarity of these scales and the recommended level, we chose to focus on the WOMAC and VAS in our analysis (Hawker et al., 1995). Different SRs/MAs have differences in literature sources, retrieval strategies, inclusion and exclusion criteria and data extraction, so it may be misleading to combine them for analysis. Therefore, we only made a descriptive analysis.

## 3 Results

### 3.1 Literature screening and selection outcomes

**FIGURE 1:**
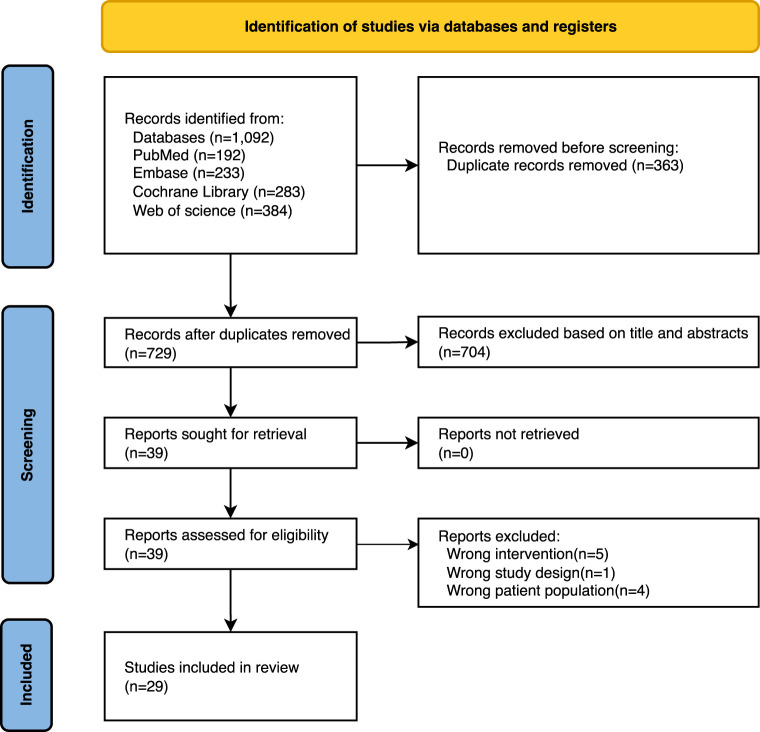
PRISMA flowchart of studies through the selection process.

The systematic search yielded 1,092 potentially eligible records. Following duplicate removal via EndNote, 729 publications remained for screening. A preliminary screening of the titles and abstracts excluded 704 obviously unqualified documents. After referring to the full text, Twenty-nine papers were finally included (Ivander and Anggono, 2024; Kim et al., 2023; Khalid et al., 2023; Vilchez-Cavazos et al., 2023; Belk et al., 2023; Idres and Samaan, 2023; Li et al., 2023; Wang et al., 2022; Peng et al., 2022; Tan et al., 2021; Gong et al., 2021; Belk et al., 2021; Nie et al., 2021; McLarnon and Heron, 2021; Filardo et al., 2021; Ren et al., 2020; Wu et al., 2020; Tang et al., 2020; Luo et al., 2020; Hohmann et al., 2020; Chen et al., 2020; Han et al., 2019; Di et al., 2018; Xu et al., 2017; Shen et al., 2017; Dai et al., 2017; Meheux et al., 2016; Kanchanatawan et al., 2016; Sadabad et al., 2016). Figure 1 presents the PRISMA flow diagram detailing the study selection process for this overview.

### 3.2 Essential features of the eligible studies

A total of 29 SRs/MAs (Ivander and Anggono, 2024; Kim et al., 2023; Khalid et al., 2023; Vilchez-Cavazos et al., 2023; Belk et al., 2023; Idres and Samaan, 2023; Li et al., 2023; Wang et al., 2022; Peng et al., 2022; Tan et al., 2021; Gong et al., 2021; Belk et al., 2021; Nie et al., 2021; McLarnon and Heron, 2021; Filardo et al., 2021; Ren et al., 2020; Wu et al., 2020; Tang et al., 2020; Luo et al., 2020; Hohmann et al., 2020; Chen et al., 2020; Han et al., 2019; Di et al., 2018; Xu et al., 2017; Shen et al., 2017; Dai et al., 2017; Meheux et al., 2016; Kanchanatawan et al., 2016; Sadabad et al., 2016) were included in the study, all of which were published between 2016 and 2024. Among the 29 SRs/MAs, the number of included original studies ranged from 4 to 42, the sample size ranged from 447 to 3,696, and all study types were randomized controlled trials comparing PRP with other agents injection. Among the methodological quality assessment tools for the included primary studies, 21 articles used the Cochrane tool only (Ivander and Anggono, 2024; Khalid et al., 2023; Vilchez-Cavazos et al., 2023; Idres and Samaan, 2023; Wang et al., 2022; Peng et al., 2022; Tan et al., 2021; Gong et al., 2021; Nie et al., 2021; McLarnon and Heron, 2021; Filardo et al., 2021; Tang et al., 2020; Luo et al., 2020; Hohmann et al., 2020; Chen et al., 2020; Han et al., 2019; Di et al., 2018; Xu et al., 2017; Shen et al., 2017; Dai et al., 2017; Kanchanatawan et al., 2016), 2 articles used the Jadad scale only (Li et al., 2023; Ren et al., 2020), 1 articles used the Modified Coleman Methodology Score (MCMS) only (Meheux et al., 2016), 2 articles used both the Cochrane tool and the Jadad scale (Wu et al., 2020; Sadabad et al., 2016), and 3 articles used both the Cochrane and the MCMS (Kim et al., 2023; Belk et al., 2023; Belk et al., 2021). WOMAC was used as the outcome measure in all of the literature. Factors such as the type and preparation method of PRP and sodium hyaluronate, injection dose, and interval were not uniformly reported in all the literature. The basic characteristics of the included studies are detailed in Table 1.

**TABLE 1:** General characteristics of included systematic reviews.

Study (year)	Included studies	Country	Total sample size	Quality evaluation tool	Intervention/comparisons	Outcomes	Protocol registered
Ivander and Anggono (2024)	4	Indonesia	447	Cochrane	PRP vs. HA	WOMAC, EQ-VAS, VAS, IKDC, Tegner	No
Kim et al. (2023)	21	South Korea	2,086	Cochrane, MCMS	PRP (LR and LP) vs. HA	WOMAC, VAS, Adverse Events	Yes
Khalid et al. (2023)	42	Pakistan/Nepal	3,696	Cochrane	PRP vs. HA, CS, placebo	WOMAC, VAS, KOOS, IKDC	Yes
Vilchez-Cavazos et al. (2023)	31	México	2,705	Cochrane	PRP vs. HA, BMAC, CS, ozone, Prolotherapy, saline	VAS, WOMAC, KOOS, IKDC	Yes
Belk et al. (2023)	27	USA	2,396	Cochrane, MCMS	PRP vs. HA, BMAC	WOMAC, VAS, KOOS, IKDC, Lequesne, Adverse Events	No
Idres and Samaan (2023)	9	Syria	608	Cochrane	PRP vs. CS	WOMAC, VAS, KOOS, IKDC, Lequesne, KSS	No
Li et al. (2023)	14	China	1,512	Jadad	multiple PRP vs. multiple HA	WOMAC, VAS, IKDC, EQ-VAS	No
Wang et al. (2022)	14	China/India	613	Cochrane	PRP vs. HA	WOMAC, EQ-VAS, IKDC, KOOS	Yes
Peng et al. (2022)	14	China/Thailand	1,485	Cochrane	PRP vs. HA	WOMAC, IKDC, VAS, Adverse Events	No
Tan et al. (2021)	26	China	2,430	Cochrane	PRP vs. HA	WOMAC, VAS, EQ-VAS, IKDC, Tegner, Lequesne, Adverse Events	No
Gong et al. (2021)	6	China	661	Cochrane	PRP vs. HA	WOMAC, IKDC, EQ-VAS, Tegner, Adverse Events	Yes
Belk et al. (2021)	18	USA	1,608	Cochrane, MCMS	PRP (LR and LP) vs. HA	WOMAC, VAS, IKDC	No
Nie et al. (2021)	21	China	1743	Cochrane	PRP (LR and LP) vs. HA, CS, saline	WOMAC, Adverse Events	Yes
McLarnon and Heron (2021)	8	UK	648	Cochrane	PRP vs. CS	WOMAC, KOOS, VAS	Yes
Filardo et al. (2021)	34	Switzerland	2,829	Cochrane	PRP vs. HA, CS, saline, ozone, prolotherapy	WOMAC, VAS, KOOS, IKDC	Yes
Ren et al. (2020)	5	China	320	Jadad	PRP vs. saline	WOMAC, VAS, IKDC	No
Wu et al. (2020)	10	China	1,063	Cochrane, Jadad	PRP vs. HA	WOMAC, IKDC, NRS, KOOS, VAS	No
Tang et al. (2020)	20	China	1,281	Cochrane	PRP vs. HA	WOMAC, IKDC, Lequesne, VAS, EQ-VAS, KOOS	No
Luo et al. (2020)	10	China	1,096	Cochrane	PRP vs. HA	WOMAC, IKDC, VAS, EQ-VAS	No
Hohmann et al. (2020)	12	South Africa/USE/Australia/USA	1,248	Cochrane	PRP vs. HA	WOMAC, IKDC, KOOS, VAS	No
Chen et al. (2020)	14	China	1,350	Cochrane	PRP vs. HA	WOMAC, VAS, IKDC, KOOS, Adverse Events	No
Han et al. (2019)	15	China	1,314	Cochrane	PRP (LR and LP) vs. HA	WOMAC, VAS, IKDC, Lequesne, Adverse Events	Yes
Di et al. (2018)	7	China	908	Cochrane	PRP vs. HA	WOMAC, IKDC, KOOS, EQ-VAS, Tegner	Yes
Xu et al. (2017)	10	China	1,184	Cochrane	PRP vs. HA, saline	WOMAC, VAS, IKDC, Lequesne	No
Shen et al. (2017)	14	China	1,423	Cochrane	PRP vs. HA, CS, saline, ozone	WOMAC, Adverse Events	Yes
Dai et al. (2017)	10	China	1,069	Cochrane	PRP vs. HA, saline	WOMAC, IKDC, Lequesne	No
Meheux et al. (2016)	6	USA	739	MCMS	PRP vs. HA, saline	VAS, IKDC, Tegner, Lequesne	Yes
Kanchanatawan et al. (2016)	9	Thailand	Not stated	Cochrane	PRP vs. HA, Placebo	WOMAC, EQ-VAS, IKDC, Lequesne	No
Sadabad et al. (2016)	6	Iran	722	Cochrane, Jadad	PRP vs. HA	WOMAC	No

BMAC, bone marrow aspirate concentrate; CS, corticosteroid; EQ-VAS, EuroQol visual analogue scale; HA, hyaluronic acid; IKDC, international knee documentation committee; KOOS, osteoarthritis outcome score; LP, leukocyte poor; LR, leukocyte rich; PRP, platelet rich plasma; MCMS, modified coleman methodology score; VAS, visual analogue scale; WOMAC, Western Ontario and McMaster Universities Osteoarthritis Index.

### 3.3 Methodological quality of included SRs/MAs

The results of the AMSTAR2 assessment showed that the methodological quality of 26 reviews (Ivander and Anggono, 2024; Kim et al., 2023; Vilchez-Cavazos et al., 2023; Belk et al., 2023; Idres and Samaan, 2023; Li et al., 2023; Wang et al., 2022; Peng et al., 2022; Tan et al., 2021; Gong et al., 2021; Belk et al., 2021; Filardo et al., 2021; Ren et al., 2020; Wu et al., 2020; Tang et al., 2020; Luo et al., 2020; Hohmann et al., 2020; Chen et al., 2020; Han et al., 2019; Di et al., 2018; Xu et al., 2017; Shen et al., 2017; Dai et al., 2017; Meheux et al., 2016; Kanchanatawan et al., 2016; Sadabad et al., 2016) was assessed as critically low quality, and 3 reviews (Khalid et al., 2023; Nie et al., 2021; McLarnon and Heron, 2021) were rated low. The main problems with the reporting of key elements were: (a) Only 12 studies reported having a written protocol and provided the registration number, but none explained the reasons for any inconsistencies with the protocol (Kim et al., 2023; Khalid et al., 2023; Vilchez-Cavazos et al., 2023; Wang et al., 2022; Gong et al., 2021; Han et al., 2019; Di et al., 2018; Shen et al., 2017; Meheux et al., 2016); (b) All studies did not involve or consult experts in the field during the search; (c) All studies did not provide a complete list of excluded studies; (d) Only 11 articles used funnel plots or statistical tests to assess publication bias (Khalid et al., 2023; Vilchez-Cavazos et al., 2023; Tan et al., 2021; Nie et al., 2021; McLarnon and Heron, 2021; Filardo et al., 2021; Luo et al., 2020; Hohmann et al., 2020; Xu et al., 2017; Dai et al., 2017; Kanchanatawan et al., 2016). Major problems with reporting of noncritical items: (a) All studies did not explain the reasons for including only the original studies of randomized controlled trials; (b) All literature did not describe the location and source of funding of the original studies; (c) Only 5 articles assessed the impact of each risk of bias on the results of the meta-analysis (Kim et al., 2023; Li et al., 2023; Nie et al., 2021; McLarnon and Heron, 2021; Dai et al., 2017) (Supplementary Table S1; Supplementary Figure S1).

### 3.4 Overlap of primary studies

Among the included studies, there were 406 nodes, with 61 having slight overlap, 31 moderate overlap, 35 high overlap, and 279 with very high overlap. The overall overlap rate was calculated at 18.42%, indicating a very high degree of overlap (Supplementary Figure S2). Risk of bias in primary studies mainly focused on deviations from intended interventions (Supplementary Figure S3).

### 3.5 Efficacy outcomes (based on SRs/MAs)

WOMAC: Four studies (Peng et al., 2022; McLarnon and Heron, 2021; Filardo et al., 2021; Tang et al., 2020) at 3 months, two studies (Filardo et al., 2021; Chen et al., 2020) at 6 months and one study (Peng et al., 2022) at 12 months showed that PRP had no significant effect on improving the WOMAC score of patients compared with the control group. On the contrary, at 3 months, six studies (Khalid et al., 2023; Li et al., 2023; Tan et al., 2021; Luo et al., 2020; Chen et al., 2020; Shen et al., 2017) showed that ten studies (Khalid et al., 2023; Li et al., 2023; Peng et al., 2022; Tan et al., 2021; Gong et al., 2021; McLarnon and Heron, 2021; Wu et al., 2020; Tang et al., 2020; Luo et al., 2020; Shen et al., 2017) at 6 months and five studies (Khalid et al., 2023; Li et al., 2023; Tan et al., 2021; Luo et al., 2020; Chen et al., 2020; Shen et al., 2017) at 12 months showed that PRP significantly improved the WOMAC score of patients (Supplementary Table S2).

VAS: At 3 months, Li et al. (2023), Peng et al. (2022) and Tang et al. (2020) found that PRP transplantation significantly improved the VAS score of patients compared with the other five studies (Khalid et al., 2023; Tan et al., 2021; McLarnon and Heron, 2021; Luo et al., 2020; Chen et al., 2020). At 6 months, two studies concluded that PRP did not improve the VAS score of patients (Khalid et al., 2023; McLarnon and Heron, 2021). At 12 months, three studies concluded that PRP did not improve patients’ VAS score (Khalid et al., 2023; Belk et al., 2021; McLarnon and Heron, 2021), and seven studies showed that PRP transplantation significantly improved patients’ VAS score (Vilchez-Cavazos et al., 2023; Belk et al., 2023; Li et al., 2023; Peng et al., 2022; Tan et al., 2021; Tang et al., 2020; Chen et al., 2020) (Supplementary Table S2).

## 4 Discussion

### 4.1 Summary of the main results

This study re-evaluated 29 SRs/MAs, published from 2016 to 2024. We adopted AMSTAR2, GROOVE and ROB2.0 to critically evaluate the SRs/MAs. AMSTAR2 assessed the methodological quality of the included studies, among which 3 studies were rated as “low quality” and 26 studies were rated as “extremely low quality.” The main defects were that the research agreement was not registered in advance, the list of excluded documents was not provided, and the original research funding source was not evaluated. These defects directly affect the credibility of the conclusion, and the curative effect may be overestimated or underestimated due to selective reporting. GROOVE tool detected a 18.42% overall overlap rate among 406 nodes, which is classified as a very high degree of overlap. With 279 nodes experiencing very high overlap, there appears to be a significant redundancy in the literature. By adopting the ROB2.0 tool, many primary studies did not implement blinding or only mentioned the use of it. Additionally, most primary studies lack explanations on outcome collection and missing data handling. We systematically summarized the published SRs/MAs, and found that extending the follow-up time from 3 months to 12 months led to the gradual improvement of the VAS score of most patients, but the WOMAC score always produced contradictory results among different SRs/MAs. Although a majority of the included SRs/MAs report trends favoring PRP over control treatments for certain outcomes and timepoints, the evidence supporting these findings is critically limited. Significant methodological flaws, very high study overlap, and risk of bias in primary studies undermine the reliability and generalizability of these reported benefits. Furthermore, inconsistency in key outcomes like WOMAC scores across reviews highlights the fragility of the evidence. Therefore, although the aggregated literature suggests a potential benefit, the low quality and inconsistency preclude confident conclusions and warrant cautious interpretation of these positive trends. Therefore, although the original data shows potential benefits, the defects in how to synthesize these evidences prevent our confidence in this trend.

### 4.2 Article limitations

(a) The study limited the search to English-language publications and conducted a manual search for certain grey literature, which may have led to the omission of relevant studies from the systematic review, thereby introducing potential bias into the research findings. (b) As there is currently no standardized agreement regarding the formulation, dosage, administration interval, and injection frequency of PRP,HA and BMAC, therefore this study does not set limits on this, and only reports and re-evaluates the research results of the original authors. (c) The vast majority of the SRs/MAs included in this study were not registered in advance on the Cochrane Collaboration Network and PROSPERO platform, lacking a certain degree of transparency and rigor, and may be subject to certain risks of bias. (d) The methodological quality assessment revealed that most individual studies included in the analyzed SRs/MAs exhibited relatively low quality scores, particularly regarding blinding procedures and allocation concealment, which may have impacted the reliability of the synthesized evidence. (e) Critical clinical stratifications including gender, racial demographics, and anthropometric measures such as body weight or BMI were absent across reviewed studies. Notably, obesity-mediated treatment response variations and implicit bias in clinical interactions may have influenced therapeutic outcomes and health disparities, potentially affecting symptom documentation and intervention timing.

### 4.3 Applicability and implications for future research

(a) Given the limited quality of existing literature, further large-scale, high-quality RCTs are needed to validate these findings and strengthen the evidence from high-quality SRs/MAs. (b) Systematic review authors should register their studies with the Cochrane Library and PROSPERO, report any protocol deviations, and critically appraise primary study methods, especially regarding standardization in PRP preparation protocols, such as leukocyte content, platelet concentration, activation, and handling. (c) Require a clear statement of conflicts of interest and independent monitoring of data analysis. Government or non-profit grants should be given priority to ensure neutrality. (d) Future studies could identify differential efficacy based on gender, race, and socioeconomic status while exploring the potential role of PRP in the continuum of treatment for KOA, particularly assessing its efficacy and cost-effectiveness after failure of short-term NSAIDs and prior to the need for surgical knee arthroplasty. (e) Establish standardized operating procedures for injection therapy for KOA, including K-L classification selection, efficacy short-term, long-term benefit and adverse event assessment.

## 5 Conclusion

In conclusion, while existing SRs/MAs frequently report positive outcomes for PRP in KOA, our evaluation found their methodological quality critically low, study overlap very high, and findings inconsistent. This severely limits the reliability of the current evidence base. Definitive conclusions regarding the efficacy and comparative effectiveness of PRP cannot be drawn. Future high-quality research, adhering rigorously to standards like AMSTAR 2, is urgently needed to provide robust evidence for clinical decision-making.

## Data Availability

The original contributions presented in the study are included in the article/Supplementary Material, further inquiries can be directed to the corresponding author.
