# Supplementary material for: Platelet rich plasma injections for knee osteoarthritis: an overview of systematic reviews
Source: Front Physiol. 2025 Jul 2;16:1598514. doi: 10.3389/fphys.2025.1598514 (PMC12263938; doi:10.3389/fphys.2025.1598514)
Supplement: Supplementary file 1 [file DataSheet1.pdf]

Supplementary Materials

eAppendix 1

The search strategy

	Query	Search term
**PubMed（192）**	#1	("Osteoarthritis, Knee"[Mesh]) OR ((((Knee Osteoarthritides[Title/Abstract]) OR (Knee Osteoarthritis[Title/Abstract])) OR (Osteoarthritis of Knee[Title/Abstract])) OR (Osteoarthritis of the Knee[Title/Abstract]))
	#2	((("Platelet-Rich Plasma"[Mesh]) OR (Platelet-Rich Plasma[Title/Abstract])) OR (Platelet Rich Plasma[Title/Abstract])) OR (Plasma, Platelet-Rich[Title/Abstract])
	#3	("Meta-Analysis as Topic"[Mesh]) OR (((Meta-Analysis[Title/Abstract]) OR (Meta analysis[Title/Abstract])) OR (systematic review[Title/Abstract]))
	#4	#1 AND #2 AND #3
**Embase（233）**	#1	TA：'knee osteoarthritis' OR 'knee osteoarthritides' OR 'osteoarthritis, knee' OR 'osteoarthritis of knee' OR 'osteoarthritis of the knee'
	#2	TA：'Platelet-Rich Plasma' OR 'Platelet-Rich Plasma' OR 'Platelet Rich Plasma' OR 'Plasma, Platelet-Rich'
	#3	TA：'systematic reviews' OR 'Meta-Analysis' OR 'Meta Analysis'
	#4	#1 AND #2 AND #3
**The Cochrane Library (283)**	#1	TAK:“Osteoarthritis, Knee” OR “Knee Osteoarthritides” OR “Knee Osteoarthritis” OR “Osteoarthritis of Knee” OR “Osteoarthritis of the Knee”
	#2	TAK: “Platelet-Rich Plasma” OR “Platelet-Rich Plasma” OR “Platelet Rich Plasma” OR “Plasma, Platelet-Rich”
	#3	TAK: “systematic reviews” OR “Meta-Analysis” OR “Meta Analysis”
	#4	#1 AND #2 AND #3
**web of science(384)**	#1	((((TS=(Osteoarthritis, Knee)) OR TS=(Knee Osteoarthritides)) OR TS=(Knee Osteoarthritis)) OR TS=(Osteoarthritis of Knee)) OR TS=(Osteoarthritis of the Knee)
	#2	(((TS=(Platelet-Rich Plasma)) OR TS=(Platelet-Rich Plasma)) OR TS=(Platelet Rich Plasma)) OR TS=(Plasma, Platelet-Rich)
	#3	((TS=(systematic review)) OR TS=(meta-analysis)) OR TS=(meta analysis)
	#4	#1 AND #2 AND #3

eTable 1

Methodological quality of included systematic reviews

Study (year)	Q1	Q2 *	Q3	Q4 *	Q5	Q6	Q7 *	Q8	Q9 *	Q10	Q11 *	Q12	Q13 *	Q14	Q15 *	Q16	Quality
Ivander et al. 2024[15]	Y	PY	N	PY	Y	Y	N	PY	Y	N	0	0	N	N	0	Y	Critically low
Kim et al. 2023[16]	Y	Y	N	PY	Y	Y	N	PY	Y	N	Y	Y	Y	Y	N	Y	Critically low
Khalid et al. 2023[17]	Y	Y	N	PY	Y	N	N	PY	Y	N	Y	N	Y	Y	Y	Y	low
Félix et al. 2023[18]	Y	Y	N	PY	Y	N	N	PY	Y	N	Y	N	N	Y	Y	Y	Critically low
Belk et al. 2023[19]	Y	N	N	PY	Y	Y	N	Y	Y	N	Y	N	PY	Y	N	Y	Critically low
Idres et al. 2023[20]	Y	PY	N	PY	Y	N	N	Y	Y	N	0	0	N	Y	0	Y	Critically low
Li et al. 2023[21]	Y	PY	N	PY	Y	Y	N	PY	Y	N	Y	Y	Y	Y	N	Y	Critically low
Wang et al. 2022[22]	Y	Y	N	PY	N	N	N	PY	Y	N	Y	N	Y	Y	N	Y	Critically low
Peng et al. 2022[23]	Y	PY	N	PY	Y	Y	N	PY	Y	N	Y	N	Y	Y	N	Y	Critically low
Tan et al. 2021[24]	Y	PY	N	PY	Y	Y	N	Y	N	N	Y	N	N	Y	Y	Y	Critically low
Gong et al. 2021[25]	Y	Y	N	PY	Y	Y	N	PY	N	N	Y	N	Y	Y	N	Y	Critically low
Belk et al. 2021[26]	Y	PY	N	PY	Y	Y	N	PY	Y	N	Y	N	N	Y	N	Y	Critically low
Nie et al. 2021[27]	Y	Y	N	PY	Y	Y	N	Y	Y	N	Y	Y	Y	Y	Y	Y	low
McLarnon et al. 2021[28]	Y	Y	N	PY	Y	Y	N	Y	Y	N	Y	Y	Y	Y	Y	Y	low
Filardo et al. 2021[29]	Y	Y	N	PY	Y	Y	N	PY	Y	N	Y	N	N	Y	Y	Y	Critically low
Ren et al. 2020[30]	Y	N	N	PY	Y	Y	N	Y	N	N	Y	N	N	Y	N	Y	Critically low
Wu et al. 2020[31]	Y	N	N	PY	Y	Y	N	PY	Y	N	Y	N	Y	Y	N	Y	Critically low
Tang et al. 2020[32]	Y	PY	N	PY	Y	Y	N	PY	Y	N	Y	N	Y	Y	N	Y	Critically low
Luo et al. 2020[33]	Y	PY	N	PY	Y	Y	N	PY	Y	N	Y	N	N	Y	Y	Y	Critically low
Hohmann et al. 2020[34]	Y	PY	N	N	Y	Y	N	Y	PY	N	N	N	N	N	Y	Y	Critically low
Chen et al. 2020[35]	Y	PY	N	PY	Y	Y	N	PY	Y	N	Y	N	Y	Y	N	Y	Critically low
Han et al. 2019[36]	Y	Y	N	PY	Y	Y	N	Y	Y	N	Y	N	N	Y	N	Y	Critically low
Di et al. 2018[37]	Y	Y	N	PY	Y	Y	N	Y	Y	N	0	0	N	N	0	Y	Critically low
Xu et al. 2017[38]	Y	PY	N	N	Y	Y	N	Y	Y	N	Y	N	Y	Y	Y	Y	Critically low
Shen et al. 2017[39]	Y	Y	N	PY	Y	Y	N	Y	Y	N	Y	N	N	N	N	Y	Critically low
Dai et al. 2017[40]	Y	PY	N	N	Y	Y	N	PY	Y	N	Y	Y	N	N	Y	Y	Critically low
Meheux et al. 2016[41]	Y	Y	N	N	Y	Y	N	Y	N	N	0	0	N	N	0	Y	Critically low
Kanchanatawan et al. 2016[42]	Y	N	N	PY	Y	Y	N	Y	PY	N	Y	N	N	Y	Y	Y	Critically low
Sadabad et al. 2016[43]	Y	PY	N	PY	Y	Y	N	PY	Y	N	Y	N	Y	Y	N	Y	Critically low

Q1-Q16 represent items 1-16 of the AMSTAR 2 evaluation list [27], *: Critical Items; Y: Yes; PY: Partial Yes; N: No; 0: No meta-analysis conducted.

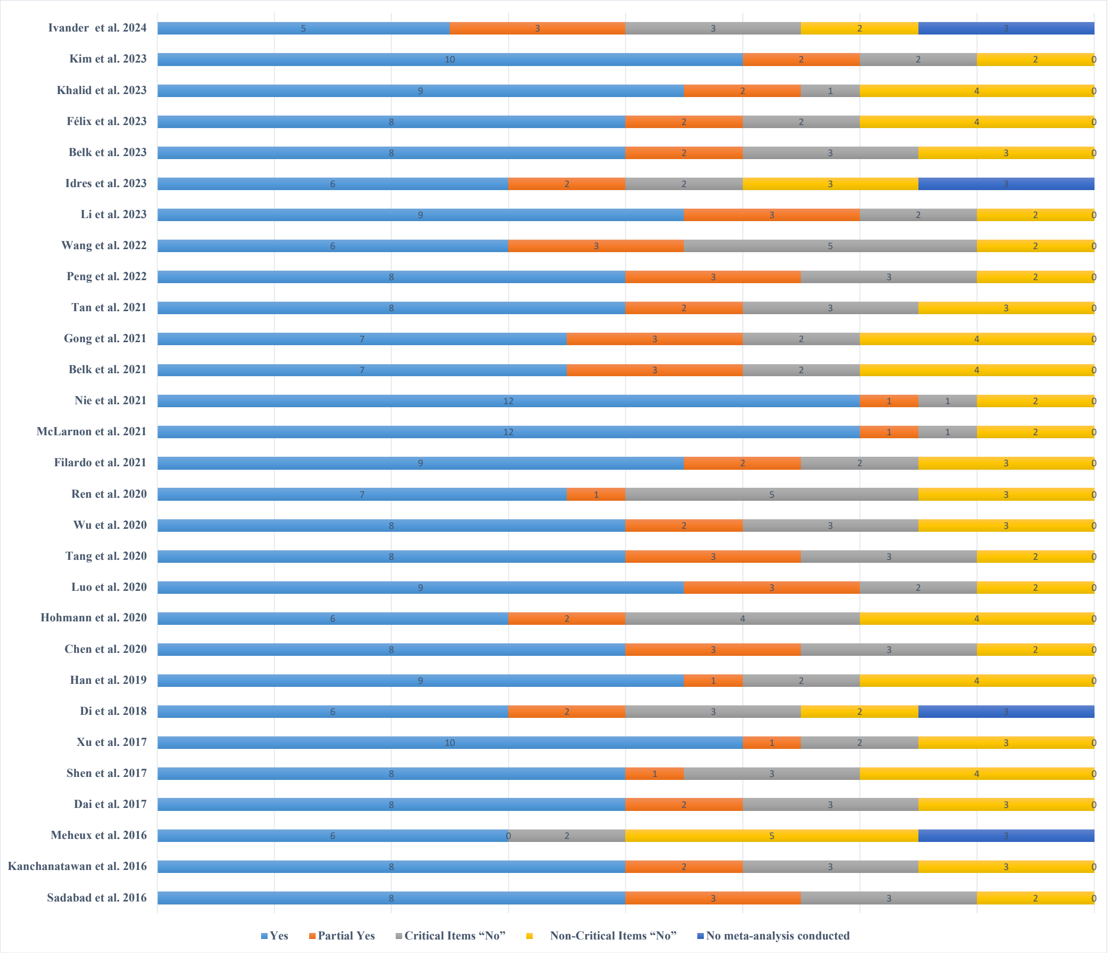

eFig.1. Overall methodological quality for each included SRs/MAs based on AMSTAR 2.

Overall results		
Number of columns (number of reviews)	c	29
Number of rows (number of index publications)	r	70
Number of included primary studies (including double counting)	N	431
Covered area	N/(rc)	21.23%
Corrected covered area	(N-r)/(rc-r)	18.42%
Interpretation of overlap	Very High overlap	
Structural Zeros	X	0
Corrected covered area (adjusting by structural zeros)	(N-r)/(rc-r-X)	18.42%
N° of non-overlapped primary studies	In 1 SR	23
Number of overlapped primary studies	In 2 SRs	5
	In 3 SRs	10
	In 4 SRs	2
	In 5 SRs	2
	In 6 SRs	2
	In 7 SRs	2
	In 8 SRs	2
	In 9 SRs	2
	In 10 SRs	1
	In 11 SRs	2
	In 12 SRs	1
	In 13 SRs	2
	In 14 SRs	0
	In 15 or more SRs	8

Graphical Representation of Overlap for OVErviews (GROOVE)

**406**	**=**	**Total nodes (pairs of reviews)**
61	=	Slight overlap (<5%)
31	=	Moderate overlap (5% to <10%)
35	=	High overlap (10% to <15%)
279	=	Very High overlap (≥15%)

	Ivander(2024)	Kim(2023)	Khalid(2023)	Félix(2023)	Belk(2023)	Idres(2023)	Li(2023)	Wang(2022)	Peng(2022)	Tan(2021)	Gong(2021)	Belk(2021)	Nie(2021)	McLarnon(2021)
Kim(2023)	8.3%													
Khalid(2023)	7.0%	39.1%												
Félix(2023)	2.9%	29.3%	46.0%											
Belk(2023)	8.0%	60.7%	47.7%	31.7%										
Idres(2023)	0.0%	3.3%	13.3%	21.2%	3.2%									
Li(2023)	12.5%	28.6%	27.3%	15.4%	42.3%	4.5%								
Wang(2022)	13.3%	40.0%	27.9%	18.9%	38.5%	0.0%	28.6%							
Peng(2022)	12.5%	33.3%	21.7%	15.4%	27.6%	0.0%	33.3%	22.7%						
Tan(2021)	11.1%	65.5%	44.7%	29.5%	58.1%	2.9%	37.9%	44.4%	48.1%					
Gong(2021)	25.0%	16.7%	11.6%	8.8%	16.0%	0.0%	33.3%	26.7%	33.3%	23.1%				
Belk(2021)	10.0%	66.7%	39.5%	28.9%	78.3%	0.0%	33.3%	47.6%	33.3%	63.0%	20.0%			
Nie(2021)	0.0%	40.0%	40.9%	37.8%	34.4%	20.8%	21.4%	17.9%	17.2%	43.8%	8.3%	35.7%		
McLarnon(2021)	0.0%	3.4%	11.1%	18.2%	3.3%	54.5%	4.8%	0.0%	0.0%	3.0%	0.0%	0.0%	16.7%	
Filardo(2021)	5.6%	47.4%	61.7%	47.7%	46.2%	13.2%	26.3%	30.6%	26.3%	57.9%	14.3%	48.6%	58.8%	10.5%
Ren(2020)	12.5%	8.0%	11.9%	5.9%	7.7%	0.0%	5.6%	12.5%	5.6%	6.9%	22.2%	9.5%	8.7%	0.0%
Wu(2020)	0.0%	24.0%	13.3%	14.3%	18.5%	0.0%	15.0%	22.2%	21.1%	25.0%	15.4%	22.7%	11.5%	0.0%
Tang(2020)	14.3%	68.0%	37.8%	27.5%	53.6%	3.6%	36.0%	37.5%	41.7%	70.4%	23.8%	58.3%	42.9%	3.7%
Luo(2020)	7.7%	45.5%	23.8%	24.2%	43.5%	0.0%	20.0%	27.8%	41.2%	38.5%	23.1%	55.6%	25.0%	0.0%
Hohmann(2020)	6.7%	41.7%	25.6%	19.4%	34.6%	0.0%	23.8%	31.6%	23.8%	40.7%	12.5%	42.9%	33.3%	0.0%
Chen(2020)	5.9%	44.0%	27.3%	21.6%	32.1%	0.0%	27.3%	35.0%	47.4%	48.1%	33.3%	39.1%	25.9%	0.0%
Han(2019)	0.0%	48.0%	32.6%	24.3%	46.2%	0.0%	20.8%	33.3%	31.8%	51.9%	16.7%	57.1%	34.6%	0.0%
Di(2018)	0.0%	26.1%	16.7%	8.6%	25.0%	0.0%	23.5%	33.3%	16.7%	26.9%	8.3%	31.6%	17.4%	0.0%
Xu(2017)	0.0%	28.0%	20.9%	17.1%	26.9%	0.0%	14.3%	21.1%	20.0%	24.1%	6.7%	33.3%	30.4%	0.0%
Shen(2017)	0.0%	38.5%	24.4%	21.6%	32.1%	4.5%	12.0%	28.6%	27.3%	33.3%	5.3%	39.1%	30.8%	0.0%
Dai(2017)	0.0%	28.0%	23.8%	20.6%	32.0%	0.0%	14.3%	21.1%	20.0%	28.6%	0.0%	40.0%	36.4%	0.0%
Meheux(2016)	0.0%	16.7%	14.3%	5.7%	16.0%	0.0%	17.6%	26.7%	11.1%	18.5%	9.1%	20.0%	23.8%	0.0%
Kanchanatawan(2016)	0.0%	24.0%	18.6%	11.1%	23.1%	0.0%	21.1%	29.4%	21.1%	25.0%	15.4%	28.6%	20.8%	0.0%
Sadabad(2016)	0.0%	21.7%	9.1%	5.7%	16.0%	0.0%	11.1%	18.8%	11.1%	18.5%	0.0%	20.0%	18.2%	0.0%

	Filardo(2021)	Ren(2020)	Wu(2020)	Tang(2020)	Luo(2020)	Hohmann(2020)	Chen(2020)	Han(2019)	Di(2018)	Xu(2017)	Shen(2017)	Dai(2017)	Meheux(2016)	Kanchanatawan(2016)
Ren(2020)	14.7%													
Wu(2020)	13.2%	7.7%												
Tang(2020)	50.0%	8.7%	16.0%											
Luo(2020)	29.4%	7.1%	18.8%	50.0%										
Hohmann(2020)	27.8%	13.3%	40.0%	33.3%	37.5%									
Chen(2020)	33.3%	5.6%	21.1%	61.9%	50.0%	23.8%								
Han(2019)	36.1%	5.3%	26.3%	59.1%	56.3%	42.1%	61.1%							
Di(2018)	17.1%	0.0%	23.1%	28.6%	30.8%	46.2%	23.5%	46.7%						
Xu(2017)	25.7%	25.0%	18.8%	36.4%	42.9%	46.7%	26.3%	47.1%	41.7%					
Shen(2017)	33.3%	18.8%	21.1%	41.7%	41.2%	44.4%	33.3%	52.6%	40.0%	71.4%				
Dai(2017)	29.4%	15.4%	11.8%	30.4%	42.9%	46.7%	20.0%	47.1%	41.7%	66.7%	60.0%			
Meheux(2016)	14.3%	10.0%	15.4%	18.2%	23.1%	38.5%	11.1%	31.3%	62.5%	45.5%	33.3%	45.5%		
Kanchanatawan(2016)	19.4%	16.7%	28.6%	26.1%	35.7%	40.0%	21.1%	41.2%	60.0%	58.3%	43.8%	46.2%	66.7%	
Sadabad(2016)	11.1%	0.0%	15.4%	18.2%	23.1%	38.5%	5.3%	23.5%	44.4%	33.3%	33.3%	33.3%	50.0%	36.4%

eFig.2. Overlapping of the included reviews

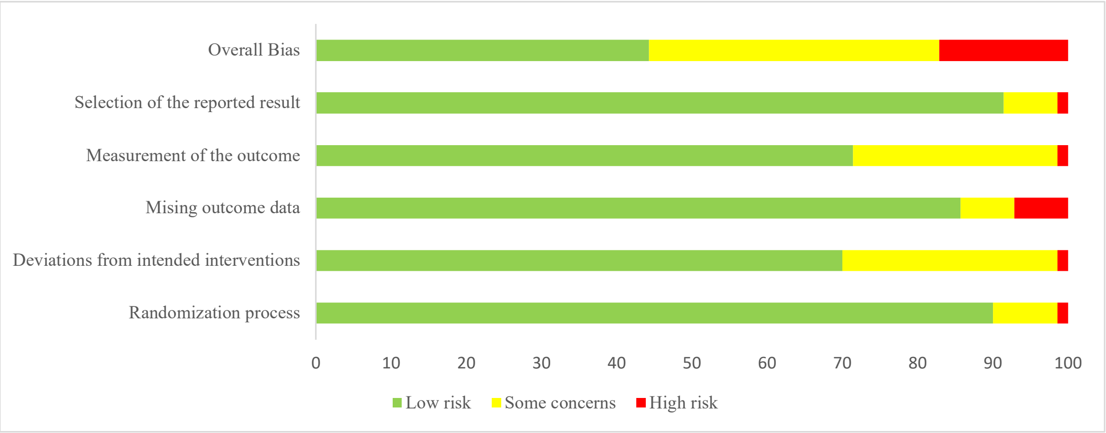

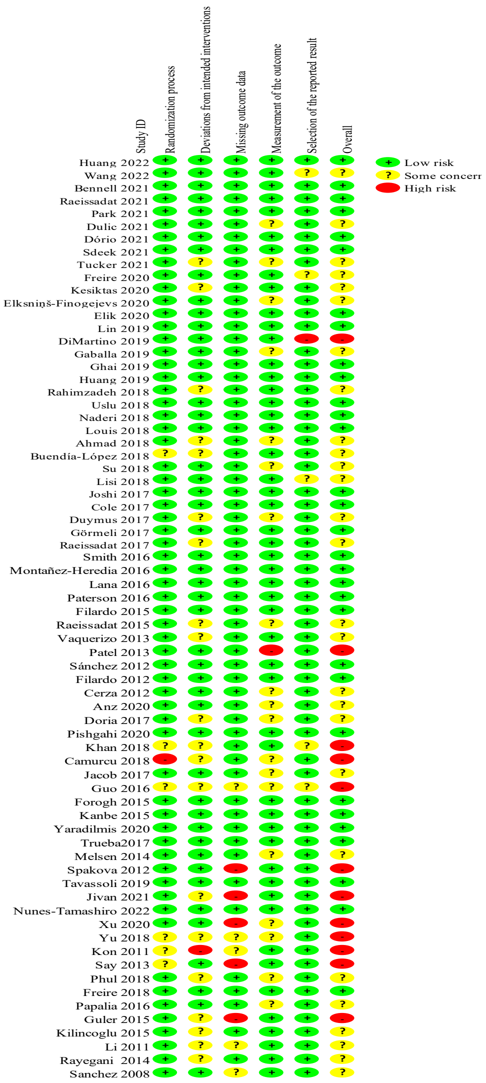

eFig.3. Risk of bias in primary study.

eTable 2

Efficacy outcomes of WOMAC and VAS

Study (year)	WOMAC 3months	WOMAC 6months	WOMAC 12 months	VAS 3 months	VAS 6 months	VAS 12 months
**Khalid et al. 2023**	WMD=-4.04;CI[-7.58,-0.49]	WMD=-6.46;CI[-9.81,-3.11]	WMD=-10.44;CI[-12.87,-8.00]	WMD=-0.55;CI[-1.30,0.21]	WMD=-0.73;CI[-1.89,0.43]	WMD=-1.06;CI[-2.39,0.28]
**Félix et al. 2023**	—	*	WMD=-1.00;CI[-1.33,-0.66]	*	*	WMD=-1.05;CI[-1.41,-0.68]
**Belk et al. 2023**	*	*	WMD=-10.88;CI[-14.02,-7.75]	*	*	WMD=-11.47;CI[-18.77,-4.16]
**Li et al. 2023**	WMD=-5.34;CI[-10.41,-0.27]	WMD=-11.02;CI[-18.09,-3.95]	WMD=-7.69;CI[-12.86,-2.52]	WMD=-0.25;CI[-0.40,-0.10]	*	WMD=-0.64;CI[-0.79,-0.49]
**Peng et al. 2022**	WMD=-2.70;CI[-7.36,1.96]	WMD=-4.29;CI[-7.66,-0.91]	WMD=-6.45;CI[-19.37,6.47]	WMD=-0.15;CI[-0.53,-0.22]	WMD=0.09;CI[-0.57,-0.75]	WMD=-0.36;CI[-1.19,-0.47]
**Tan et al. 2021**	WMD=-5.04;CI[-8.82,-1.26]	WMD=-8.52;CI[-11.17,-5.87]	WMD=-10.52;CI[-13.77,-7.27]	WMD=-0.47;CI[-1.04,0.05]	WMD=-0.69;CI[-1.33,-0.05]	WMD=-0.99;CI[-1.54,-0.45]
**Gong et al. 2021**	*	WMD=-4.79;CI[-4.00,-5.59]	WMD=-3.85;CI[-2.66,-5.04]	*	*	*
**Belk et al. 2021**	*	*	WMD=-13.63;CI[-18.17,-9.09]	*	*	WMD=-9.80;CI[-20.48,0.89]
**McLarnon et al. 2021**	WMD=-2.15;CI[-5.65,1.35]	WMD=-9.51;CI[-15.20,-3.83]	WMD=-8.10;CI[-23.86,-7.65]	WMD=-0.51;CI[-1.02,0.01]	WMD=-0.97;CI[-1.94,0.00]	WMD=-0.07;CI[-0.42,0.28]
**Filardo et al. 2021**	WMD=-10.71;CI[-23.71,2.29]	WMD=-12.50;CI[-25.69,0.69]	WMD=-19.38;CI[-36.04,-2.72]	*	*	*
**Wu et al. 2020**	*	WMD=-20.69;CI[-24.50,-16.89]	*	*	WMD=-1.50;CI[-1.61,-1.38]	*
**Tang et al. 2020**	WMD=-0.13;CI[-0.78,0.52]	WMD=-1.14;CI[-1.88,-0.40]	WMD=-1.47;CI[-2.23,-0.70]	WMD=-0.54;CI[-1.03,-0.05]	WMD=-0.77;CI[-1.24,-0.29]	WMD=-0.99;CI[-1.54,-0.45]
**Luo et al. 2020**	WMD=-1.35;CI[-2.19,-0.50]	WMD=-3.89;CI[-5.60,-2.18]	WMD=-8.79;CI[-16.22,-1.35]	WMD=-0.35;CI[-1.23,0.54]	WMD=-1.27;CI[-2.36,-0.18]	*
**Chen et al. 2020**	WMD=-0.59;CI[-0.96,-0.23]	WMD=-0.35;CI[-0.64,0.07]	WMD=-0.50;CI[-0.67,-0.33]	WMD=0.04;CI[-0.29,0.37]	WMD=-0.36;CI[-0.67,-0.20]	WMD=-0.04;CI[-0.67,-0.20]
**Shen et al. 2017**	WMD=-14.53;CI[-21.97,-7.09]	WMD=-18.21;CI[-27.84,-8.59]	WMD=-19.45;CI[-26.09,-12.82]	*	*	*

References

1. Huang HY, Hsu CW, Lin GC, et al. Comparing efficacy of a single intraarticular injection of platelet-rich plasma (PRP) combined with different hyaluronans for knee osteoarthritis: a randomized-controlled clinical trial. *BMC Musculoskelet Disord*. 2022;23(1):954. Published 2022 Nov 4. doi:10.1186/s12891-022-05906-5
2. Wang YC, Lee CL, Chen YJ, et al. Comparing the Efficacy of Intra-Articular Single Platelet-Rich Plasma(PRP) versus Novel Crosslinked Hyaluronic Acid for Early-Stage Knee Osteoarthritis: A Prospective, Double-Blind, Randomized Controlled Trial. *Medicina (Kaunas)*. 2022;58(8):1028. Published 2022 Aug 1. doi:10.3390/medicina58081028
3. Bennell KL, Paterson KL, Metcalf BR, et al. Effect of Intra-articular Platelet-Rich Plasma vs Placebo Injection on Pain and Medial Tibial Cartilage Volume in Patients With Knee Osteoarthritis: The RESTORE Randomized Clinical Trial. *JAMA*. 2021;326(20):2021-2030. doi:10.1001/jama.2021.19415
4. Raeissadat SA, Ghazi Hosseini P, Bahrami MH, et al. The comparison effects of intra-articular injection of Platelet Rich Plasma (PRP), Plasma Rich in Growth Factor (PRGF), Hyaluronic Acid (HA), and ozone in knee osteoarthritis; a one year randomized clinical trial. *BMC Musculoskelet Disord*. 2021;22(1):134. Published 2021 Feb 3. doi:10.1186/s12891-021-04017-x
5. Park YB, Kim JH, Ha CW, Lee DH. Clinical Efficacy of Platelet-Rich Plasma Injection and Its Association With Growth Factors in the Treatment of Mild to Moderate Knee Osteoarthritis: A Randomized Double-Blind Controlled Clinical Trial As Compared With Hyaluronic Acid. *Am J Sports Med*. 2021;49(2):487-496. doi:10.1177/0363546520986867
6. Dulic O, Rasovic P, Lalic I, et al. Bone Marrow Aspirate Concentrate versus Platelet Rich Plasma or Hyaluronic Acid for the Treatment of Knee Osteoarthritis. *Medicina (Kaunas)*. 2021;57(11):1193. Published 2021 Nov 2. doi:10.3390/medicina57111193
7. Dório M, Pereira RMR, Luz AGB, Deveza LA, de Oliveira RM, Fuller R. Efficacy of platelet-rich plasma and plasma for symptomatic treatment of knee osteoarthritis: a double-blinded placebo-controlled randomized

clinical trial. *BMC Musculoskelet Disord*. 2021;22(1):822. Published 2021 Sep 24. doi:10.1186/s12891-021-04706-7

8. Sdeek M, Sabry D, El-Sdeek H, Darweash A. Intra-articular injection of Platelet rich plasma versus Hyaluronic acid for moderate knee osteoarthritis. A prospective, double-blind randomized controlled trial on 189 patients with follow-up for three years. *Acta Orthop Belg*. 2021;87(4):729-734. doi:10.52628/87.4.18
9. Tucker JD, Goetz LL, Duncan MB, et al. Randomized, Placebo-Controlled Analysis of the Knee Synovial Environment Following Platelet-Rich Plasma Treatment for Knee Osteoarthritis. *PM R*. 2021;13(7):707-719. doi:10.1002/pmrj.12561
10. Freire MRM, da Silva PMC, Azevedo AR, Silva DS, da Silva RBB, Cardoso JC. Comparative Effect between Infiltration of Platelet-rich Plasma and the Use of Corticosteroids in the Treatment of Knee Osteoarthritis: A Prospective and Randomized Clinical Trial. *Rev Bras Ortop (Sao Paulo)*. 2020;55(5):551-556. doi:10.1016/j.rbo.2018.01.001
11. Kesiktas FN, Dernek B, Sen EI, Albayrak HN, Aydin T, Yildiz M. Comparison of the short-term results of single-dose intra-articular peptide with hyaluronic acid and platelet-rich plasma injections in knee osteoarthritis: a randomized study. *Clin Rheumatol*. 2020;39(10):3057-3064. doi:10.1007/s10067-020-05121-4
12. Elksniņš-Finogejevs A, Vidal L, Peredistijs A. Intra-articular platelet-rich plasma vs corticosteroids in the treatment of moderate knee osteoarthritis: a single-center prospective randomized controlled study with a 1-year follow up. *J Orthop Surg Res*. 2020;15(1):257. Published 2020 Jul 10. doi:10.1186/s13018-020-01753-z
13. Elik H, Doğu B, Yılmaz F, Begoğlu FA, Kuran B. The efficiency of platelet-rich plasma treatment in patients with knee osteoarthritis. *J Back Musculoskelet Rehabil*. 2020;33(1):127-138. doi:10.3233/BMR-181374
14. Lin KY, Yang CC, Hsu CJ, Yeh ML, Renn JH. Intra-articular Injection of Platelet-Rich Plasma Is Superior to Hyaluronic Acid or Saline Solution in the Treatment of Mild to Moderate Knee Osteoarthritis: A Randomized, Double-Blind, Triple-Parallel, Placebo-Controlled Clinical Trial. *Arthroscopy*. 2019;35(1):106-117. doi:10.1016/j.arthro.2018.06.035
15. Di Martino A, Di Matteo B, Papio T, et al. Platelet-Rich Plasma Versus Hyaluronic Acid Injections for the Treatment of Knee Osteoarthritis: Results at 5 Years of a Double-Blind, Randomized Controlled Trial. *Am J Sports Med*. 2019;47(2):347-354. doi:10.1177/0363546518814532
16. Gaballa NM, Mohammed YA, Kamel LM, Mahgoub HM (2019) Therapeutic efficacy of intra-articular injection of platelet–rich plasma and ozone therapy in patients with primary knee osteoarthritis. Egypt Rheumatol2019;41(7):183–187.https://doi.org/10.1016/j.ejr.2018.07.005
17. Ghai B, Gupta V, Jain A, Goel N, Chouhan D, Batra YK. Efetividade do plasma rico em plaquetas no tratamento da dor em osteoartrite de joelho: estudo comparativo randômico e duplo-cego [Effectiveness of platelet rich plasma in pain management of osteoarthritis knee: double blind, randomized comparative study]. *Braz J Anesthesiol*. 2019;69(5):439-447. doi:10.1016/j.bjan.2019.06.003
18. Huang Y, Liu X, Xu X, Liu J. Intra-articular injections of platelet-rich plasma, hyaluronic acid or corticosteroids for knee osteoarthritis : A prospective randomized controlled study. Intraartikuläre Injektionen mit plättchenreichem Plasma, Hyaluronsäure oder Kortikosteroiden bei Kniearthrose : Eine prospektive, randomisierte, kontrollierte Studie. *Orthopade*. 2019;48(3):239-247. doi:10.1007/s00132-018-03659-5
19. Rahimzadeh P, Imani F, Faiz SHR, Entezary SR, Zamanabadi MN, Alebouyeh MR. The effects of injecting intra-articular platelet-rich plasma or prolotherapy on pain score and function in knee osteoarthritis. *Clin Interv Aging*. 2018;13:73-79. Published 2018 Jan 4. doi:10.2147/CIA.S147757

20. Uslu Güvendi E, Aşkin A, Güvendi G, Koçyiğit H. Comparison of Efficiency Between Corticosteroid and Platelet Rich Plasma Injection Therapies in Patients With Knee Osteoarthritis. *Arch Rheumatol*. 2017;33(3):273-281. Published 2017 Nov 2. doi:10.5606/ArchRheumatol.2018.6608
21. Nabi B N , Sedighinejad A , Mardanikivi M ,et al.Comparing the Effectiveness of Intra-articular Platelet-Rich Plasma and Corticosteroid Injection under Ultrasound Guidance on Pain Control of Knee Osteoarthritis.2018;20:e62157.DOI:10.5812/IRCMJ.62157.
22. Louis ML, Magalon J, Jouve E, et al. Growth Factors Levels Determine Efficacy of Platelets Rich Plasma Injection in Knee Osteoarthritis: A Randomized Double Blind Noninferiority Trial Compared With Viscosupplementation. *Arthroscopy*. 2018;34(5):1530-1540.e2. doi:10.1016/j.arthro.2017.11.035
23. Ahmad HS, Farrag SE, Okasha AE, et al. Clinical outcomes are associated with changes in ultrasonographic structural appearance after platelet-rich plasma treatment for knee osteoarthritis. *Int J Rheum Dis*. 2018;21(5):960-966. doi:10.1111/1756-185X.13315
24. Buendía-López D, Medina-Quirós M, Fernández-Villacañas Marín MÁ. Clinical and radiographic comparison of a single LP-PRP injection, a single hyaluronic acid injection and daily NSAID administration with a 52-week follow-up: a randomized controlled trial. *J Orthop Traumatol*. 2018;19(1):3. Published 2018 Aug 20. doi:10.1186/s10195-018-0501-3
25. Su K, Bai Y, Wang J, Zhang H, Liu H, Ma S. Comparison of hyaluronic acid and PRP intra-articular injection with combined intra-articular and intraosseous PRP injections to treat patients with knee osteoarthritis. *Clin Rheumatol*. 2018;37(5):1341-1350. doi:10.1007/s10067-018-3985-6
26. Lisi C, Perotti C, Scudeller L, et al. Treatment of knee osteoarthritis: platelet-derived growth factors vs. hyaluronic acid. A randomized controlled trial. *Clin Rehabil*. 2018;32(3):330-339. doi:10.1177/0269215517724193
27. Joshi Jubert N, Rodríguez L, Reverté-Vinaixa MM, Navarro A. Platelet-Rich Plasma Injections for Advanced Knee Osteoarthritis: A Prospective, Randomized, Double-Blinded Clinical Trial. *Orthop J Sports Med*. 2017;5(2):2325967116689386. Published 2017 Feb 13. doi:10.1177/2325967116689386
28. Cole BJ, Karas V, Hussey K, Pilz K, Fortier LA. Hyaluronic Acid Versus Platelet-Rich Plasma: A Prospective, Double-Blind Randomized Controlled Trial Comparing Clinical Outcomes and Effects on Intra-articular Biology for the Treatment of Knee Osteoarthritis [published correction appears in Am J Sports Med. 2017 Apr;45(5):NP10. doi: 10.1177/0363546517700110.]. *Am J Sports Med*. 2017;45(2):339-346. doi:10.1177/0363546516665809
29. Duymus TM, Mutlu S, Dernek B, Komur B, Aydogmus S, Kesiktas FN. Choice of intra-articular injection in treatment of knee osteoarthritis: platelet-rich plasma, hyaluronic acid or ozone options. *Knee Surg Sports Traumatol Arthrosc*. 2017;25(2):485-492. doi:10.1007/s00167-016-4110-5
30. Görmeli G, Görmeli CA, Ataoglu B, Çolak C, Aslantürk O, Ertem K. Multiple PRP injections are more effective than single injections and hyaluronic acid in knees with early osteoarthritis: a randomized, double-blind, placebo-controlled trial. *Knee Surg Sports Traumatol Arthrosc*. 2017;25(3):958-965. doi:10.1007/s00167-015-3705-6
31. Raeissadat SA, Rayegani SM, Ahangar AG, Abadi PH, Mojgani P, Ahangar OG. Efficacy of Intra-articular Injection of a Newly Developed Plasma Rich in Growth Factor (PRGF) Versus Hyaluronic Acid on Pain and Function of Patients with Knee Osteoarthritis: A Single-Blinded Randomized Clinical Trial. *Clin Med Insights Arthritis Musculoskelet Disord*. 2017;10:1179544117733452. Published 2017 Oct 10. doi:10.1177/1179544117733452

32. Smith PA. Intra-articular Autologous Conditioned Plasma Injections Provide Safe and Efficacious Treatment for Knee Osteoarthritis: An FDA-Sanctioned, Randomized, Double-blind, Placebo-controlled Clinical Trial. *Am J Sports Med*. 2016;44(4):884-891. doi:10.1177/0363546515624678
33. Montañez-Heredia E, Irízar S, Huertas PJ, et al. Intra-Articular Injections of Platelet-Rich Plasma versus Hyaluronic Acid in the Treatment of Osteoarthritic Knee Pain: A Randomized Clinical Trial in the Context of the Spanish National Health Care System. *Int J Mol Sci*. 2016;17(7):1064. Published 2016 Jul 2. doi:10.3390/ijms17071064
34. Lana JF, Weglein A, Sampson SE, et al. Randomized controlled trial comparing hyaluronic acid, platelet-rich plasma and the combination of both in the treatment of mild and moderate osteoarthritis of the knee. *J Stem Cells Regen Med*. 2016;12(2):69-78. Published 2016 Nov 29. doi:10.46582/jsrm.1202011
35. Paterson KL, Nicholls M, Bennell KL, Bates D. Intra-articular injection of photo-activated platelet-rich plasma in patients with knee osteoarthritis: a double-blind, randomized controlled pilot study. *BMC Musculoskelet Disord*. 2016;17:67. Published 2016 Feb 9. doi:10.1186/s12891-016-0920-3
36. Filardo G, Di Matteo B, Di Martino A, et al. Platelet-Rich Plasma Intra-articular Knee Injections Show No Superiority Versus Viscosupplementation: A Randomized Controlled Trial. *Am J Sports Med*. 2015;43(7):1575-1582. doi:10.1177/0363546515582027
37. Raeissadat SA, Rayegani SM, Hassanabadi H, et al. Knee Osteoarthritis Injection Choices: Platelet- Rich Plasma (PRP) Versus Hyaluronic Acid (A one-year randomized clinical trial). *Clin Med Insights Arthritis Musculoskelet Disord*. 2015;8:1-8. Published 2015 Jan 7. doi:10.4137/CMAMD.S17894
38. Vaquerizo V, Plasencia MÁ, Arribas I, et al. Comparison of intra-articular injections of plasma rich in growth factors (PRGF-Endoret) versus Durolane hyaluronic acid in the treatment of patients with symptomatic osteoarthritis: a randomized controlled trial [published correction appears in Arthroscopy. 2024 Jun;40(6):1953. doi: 10.1016/j.arthro.2024.03.034.]. *Arthroscopy*. 2013;29(10):1635-1643. doi:10.1016/j.arthro.2013.07.264
39. Patel S, Dhillon MS, Aggarwal S, Marwaha N, Jain A. Treatment with platelet-rich plasma is more effective than placebo for knee osteoarthritis: a prospective, double-blind, randomized trial. *Am J Sports Med*. 2013;41(2):356-364. doi:10.1177/0363546512471299
40. Sánchez M, Fiz N, Azofra J, et al. A randomized clinical trial evaluating plasma rich in growth factors (PRGF-Endoret) versus hyaluronic acid in the short-term treatment of symptomatic knee osteoarthritis. *Arthroscopy*. 2012;28(8):1070-1078. doi:10.1016/j.arthro.2012.05.011
41. Filardo G, Kon E, Di Martino A, et al. Platelet-rich plasma vs hyaluronic acid to treat knee degenerative pathology: study design and preliminary results of a randomized controlled trial. *BMC Musculoskelet Disord*. 2012;13:229. Published 2012 Nov 23. doi:10.1186/1471-2474-13-229
42. Cerza F, Carnì S, Carcangiu A, et al. Comparison between hyaluronic acid and platelet-rich plasma, intra-articular infiltration in the treatment of gonarthrosis. *Am J Sports Med*. 2012;40(12):2822-2827. doi:10.1177/0363546512461902
43. Anz AW, Hubbard R, Rendos NK, Everts PA, Andrews JR, Hackel JG. Bone Marrow Aspirate Concentrate Is Equivalent to Platelet-Rich Plasma for the Treatment of Knee Osteoarthritis at 1 Year: A Prospective, Randomized Trial. *Orthop J Sports Med*. 2020;8(2):2325967119900958. Published 2020 Feb 18. doi:10.1177/2325967119900958
44. Doria C, Mosele GR, Caggiari G, Puddu L, Ciurlia E. Treatment of Early Hip Osteoarthritis: Ultrasound-Guided Platelet Rich Plasma versus Hyaluronic Acid Injections in a Randomized Clinical Trial. *Joints*. 2017;5(3):152-155. Published 2017 Aug 11. doi:10.1055/s-0037-1605584

45. Pishgahi A, Abolhasan R, Shakouri SK, et al. Effect of Dextrose Prolotherapy, Platelet Rich Plasma and Autologous Conditioned Serum on Knee Osteoarthritis: A Randomized Clinical Trial. *Iran J Allergy Asthma Immunol*. 2020;19(3):243-252. Published 2020 Jun 23. doi:10.18502/ijaai.v19i3.3452
46. Khan AF, Gillani SFUHS, Khan AF. Role of intra-articular corticosteroid with xylocaine vs plate rich plasma for the treatment of early grade II knee osteoarthritis at Akhtar Saeed Teaching Hospital Lahore: a randomized controlled trail. Pakistan J Med Health Sci 2018;12:1432–1435.
47. Camurcu Y, Sofu H, Ucpunar H, Kockara N, Cobden A, Duman S. Single-dose intra-articular corticosteroid injection prior to platelet-rich plasma injection resulted in better clinical outcomes in patients with knee osteoarthritis: A pilot study. *J Back Musculoskelet Rehabil*. 2018;31(4):603-610. doi:10.3233/BMR-171066
48. Jacob G, Shetty V, Shetty S (2017) A study assessing intra-artic- ular PRP vs PRP with HMW HA vs PRP with LMW HA in early knee osteoarthritis. J Arthrosc Jt Surg 4:65–71. https://doi.org/10.1016/j.jajs.2017.08.008
49. Guo Y, Yu H, Yuan L et al (2016) Treatment of knee osteoarthritis with platelet-rich plasma plus hyaluronic acid in comparison with platelet-rich plasma only. Int J Clin Exp Med 9:12085–12090
50. Forogh B, Mianehsaz E, Shoaee S, Ahadi T, Raissi GR, Sajadi S. Effect of single injection of platelet-rich plasma in comparison with corticosteroid on knee osteoarthritis: a double-blind randomized clinical trial. *J Sports Med Phys Fitness*. 2016;56(7-8):901-908.
51. Kanbe K, Chiba J, Inoue Y, Taguchi M, Yabuki A. Predictive factors related to the efficacy of golimumab in patients with rheumatoid arthritis. *Clin Med Insights Arthritis Musculoskelet Disord*. 2015;8:25-32. Published 2015 Feb 19. doi:10.4137/CMAMD.S22155
52. Yaradilmis YU, Demirkale I, Safa Tagral A, Caner Okkaoglu M, Ates A, Altay M. Comparison of two platelet rich plasma formulations with viscosupplementation in treatment of moderate grade gonarthrosis: A prospective randomized controlled study. *J Orthop*. 2020;20:240-246. Published 2020 Jan 28. doi:10.1016/j.jor.2020.01.041
53. Trueba Vasavilbaso C, Rosas Bello CD, Medina López E, et al. Benefits of different postoperative treatments in patients undergoing knee arthroscopic debridement. *Open Access Rheumatol*. 2017;9:171-179. Published 2017 Sep 25. doi:10.2147/OARRR.S138353
54. Melsen WG, Bootsma MC, Rovers MM, Bonten MJ (2014) The effects of clinical and statistical heterogeneity on the predic- tive values of results from meta-analyses. Clin Microbiol Infect 20:123–129. https://doi.org/10.1111/1469-0691.12494
55. Spaková T, Rosocha J, Lacko M, Harvanová D, Gharaibeh A. Treatment of knee joint osteoarthritis with autologous platelet-rich plasma in comparison with hyaluronic acid. *Am J Phys Med Rehabil*. 2012;91(5):411-417. doi:10.1097/PHM.0b013e3182aab72
56. Tavassoli M, Janmohammadi N, Hosseini A, Khafri S, Esmaeilnejad-Ganji SM. Single- and double-dose of platelet-rich plasma *versus* hyaluronic acid for treatment of knee osteoarthritis: A randomized controlled trial. *World J Orthop*. 2019;10(9):310-326. Published 2019 Sep 18. doi:10.5312/wjo.v10.i9.310
57. Jalali Jivan S, Monzavi SM, Zargaran B, et al. Comparative Analysis of the Effectiveness of Intra-Articular Injection of Platelet-Rich Plasma versus Hyaluronic Acid for Knee Osteoarthritis: Results of an Open-Label Trial. *Arch Bone Jt Surg*. 2021;9(5):487-495. doi:10.22038/abjs.2021.52003.2569
58. Nunes-Tamashiro JC, Natour J, Ramuth FM, et al. Intra-articular injection with platelet-rich plasma compared to triamcinolone hexacetonide or saline solution in knee osteoarthritis: A double blinded randomized controlled trial with one year follow-up. *Clin Rehabil*. 2022;36(7):900-915. doi:10.1177/02692155221090407
59. Xu Z, He Z, Shu L, Li X, Ma M, Ye C. Intra-Articular Platelet-Rich Plasma Combined With Hyaluronic Acid Injection for Knee Osteoarthritis Is Superior to Platelet-Rich Plasma or Hyaluronic Acid Alone in Inhibiting

Inflammation and Improving Pain and Function. *Arthroscopy*. 2021;37(3):903-915. doi:10.1016/j.arthro.2020.10.013

60. Yu W, Xu P, Huang G, Liu L. Clinical therapy of hyaluronic acid combined with platelet-rich plasma for the treatment of knee osteoarthritis. *Exp Ther Med*. 2018;16(3):2119-2125. doi:10.3892/etm.2018.6412
61. Kon E, Mandelbaum B, Buda R, et al. Platelet-rich plasma intra-articular injection versus hyaluronic acid viscosupplementation as treatments for cartilage pathology: from early degeneration to osteoarthritis. *Arthroscopy*. 2011;27(11):1490-1501. doi:10.1016/j.arthro.2011.05.011
62. Say F, Gürler D, Yener K, Bülbül M, Malkoc M. Platelet-rich plasma injection is more effective than hyaluronic acid in the treatment of knee osteoarthritis. *Acta Chir Orthop Traumatol Cech*. 2013;80(4):278-283.
63. Phul SH, Mobushir M, Jilani RUA, Khan IS, Malik RH, Jan G. Comparison of Intra-Articular Steroids Injection Versus Platelets Rich Plasma Injection in Patients with Osteoarthritic Knee Joints; 2018.
64. de Menezes Freire MR, da Silva PMC, Azevedo AR, Silva DS, da Silva RBB, Cardoso JC. Efeito comparativo entre a infiltração de plasma rico em plaquetas e o uso de corti- costeroides no tratamento de osteoartrite do joelho: estudo clínico prospectivo e randomizado. Rev Bras Ortop. 2018. doi:10.1016/j.rbo.2018.01.001
65. Papalia R, Zampogna B, Russo F, et al. Comparing hybrid hyaluronic acid with PRP in end career athletes with degenerative cartilage lesions of the knee. *J Biol Regul Homeost Agents*. 2016;30(4 Suppl 1):17-23.
66. Guler O, Mutlu S, Isyar M, Seker A, Kayaalp ME, Mahirogullari M. Comparison of short-term results of intraarticular platelet-rich plasma (PRP) and hyaluronic acid treatments in early-stage gonarthrosis patients. *Eur J Orthop Surg Traumatol*. 2015;25(3):509-513. doi:10.1007/s00590-014-1517-x
67. Kilincoglu V, Yeter A, Servet E, Kangal M, Yildirim M. Short term results comparison of intraarticular platelet-rich plasma (prp) and hyaluronic acid (ha) applications in early stage of knee osteoarthritis. *Int J Clin Exp Med*. 2015;8(10):18807-18812. Published 2015 Oct 15.
68. Li M, Zhang C, Ai Z, Yuan T, Feng Y, Jia W. *Zhongguo Xiu Fu Chong Jian Wai Ke Za Zhi*. 2011;25(10):1192-1196.
69. Rayegani SM, Raeissadat SA, Taheri MS, et al. Does intra articular platelet rich plasma injection improve function, pain and quality of life in patients with osteoarthritis of the knee? A randomized clinical trial. *Orthop Rev (Pavia)*. 2014;6(3):5405. Published 2014 Sep 18. doi:10.4081/or.2014.5405
70. Sánchez M, Anitua E, Azofra J, Aguirre JJ, Andia I. Intra-articular injection of an autologous preparation rich in growth factors for the treatment of knee OA: a retrospective cohort study. *Clin Exp Rheumatol*. 2008;26(5):910-913.
